# The Meniscus: Basic Science and Therapeutic Approaches

**DOI:** 10.3390/jcm14062020

**Published:** 2025-03-16

**Authors:** Nikodem Kuczyński, Julia Boś, Kinga Białoskórska, Zuzanna Aleksandrowicz, Bartosz Turoń, Maria Zabrzyńska, Klaudia Bonowicz, Maciej Gagat

**Affiliations:** 1 Department of Morphological and Physiological Sciences, Faculty of Medicine, Collegium Medicum, Mazovian Academy in Płock, 09-402 Płock, Poland; n.kuczynski@mazowiecka.edu.pl (N.K.); j.bos@mazowiecka.edu.pl (J.B.); kingabialoskorska@gmail.com (K.B.); ale.zuz@wp.pl (Z.A.); klaudia.bonowicz@cm.umk.pl (K.B.); 2 Department of Trauma and Orthopedics, Regional Specialist Hospital in Grudziądz, 86-300 Grudziądz, Poland; b.turon@medipunkt.pl; 3 Department of Family Medicine, Faculty of Medicine, Collegium Medicum in Bydgoszcz, Nicolaus Copernicus University in Toruń, 85-094 Bydgoszcz, Poland; maria.zabrzynska@cm.umk.pl; 4 Department of Histology and Embryology, Faculty of Medicine, Collegium Medicum in Bydgoszcz, Nicolaus Copernicus University in Toruń, 85-092 Bydgoszcz, Poland

**Keywords:** meniscus, osteoarthritis, knee biomechanics, regenerative therapies

## Abstract

The proper function and longevity of the knee joint are ensured by the knee menisci. Their susceptibility to damage and injury is one of the main risk factors for rapid cartilage loss and the development of osteoarthritis. The vascularization pattern and nutritional status of a torn meniscus determine its potential for healing and the success of meniscus surgery. Blood supply is a crucial factor in assessing healing potential. Knee cartilage volume loss and its modification often result from meniscal damage or excision, leading to osteoarthritis. Modern methods for preserving meniscal tissue are currently the treatment of choice. Magnetic resonance imaging (MRI) is the gold standard for assessing meniscus lesions. It provides a comprehensive evaluation of tear stability and progression risk. Additionally, it offers high sensitivity and specificity. Arthrography combined with computed tomography (CT) can be used for patients who are unable to undergo MRI. Other methods, such as X-ray and ultrasound, are not useful for the typical diagnosis of meniscal lesions. Minimally invasive surgery has become the gold standard for both treatment and diagnosis. Modern techniques, such as all-inside compression sutures and other suturing techniques, are also considered. In contrast, in the past, open total meniscectomy was routinely performed as the gold standard, based on the mistaken belief that the menisci were functionless. Currently, new treatment methods for meniscal lesions are being explored, including mesenchymal stem cells, synthetic implants, and platelet-rich plasma (PRP). The crucial role of the menisci in knee biomechanics drives the development of modern solutions focused on preserving meniscal tissue.

## 1. Introduction

The menisci of the knee are recognized as essential structures for the proper function and longevity of the knee joint. Although, in the past, they were considered embryonic remnants, today their role is well understood. The menisci are involved in several key biomechanical functions. They distribute load across the tibiofemoral joint, reducing stress on the articular cartilage. Additionally, they provide a contact surface, guide rotation, and stable translation [1,2]. The medial and lateral menisci are fibrous–cartilaginous structures that are composed of a well-organized network of collagen, proteoglycans, glycoproteins, and other cellular components. The menisci are essential for preserving the integrity of the knee’s articular surfaces during weight-bearing activities [3]. On the other hand, their complex structure makes them vulnerable to damage and injuries. Meniscal tears are frequent and become more prevalent with age. Activities such as squatting, kneeling, crawling, prolonged sitting while driving, stair climbing, lifting objects, and walking are linked to acute meniscal tears. Meniscal tears can present with various morphological patterns, each associated with different causes and injury mechanisms. A primary distinction is made between acute and degenerative tears. Acute tears typically result from trauma or sports-related injuries, whereas degenerative tears develop due to aging, chronic joint instability, and malalignment. When determining treatment, factors such as the shape, structure, and location of the tear must be taken into account. The most frequently observed tear patterns include horizontal tears, bucket handle tears, longitudinal tears, oblique (flap) tears, radial tears, meniscal root tears, and complex tears, which involve a combination of different tear types [4].

Meniscal damage is one of the main risk factors for rapid cartilage degeneration [5]. Injuries to the meniscus can also cause mechanical issues in the knee, such as pain, joint locking, and swelling. When meniscal function is compromised, it can accelerate the onset of osteoarthritis, which is particularly concerning for younger, active individuals [3]. Osteoarthritis (OA) is a leading cause of disability worldwide, particularly among aging populations. The primary symptoms of OA include joint pain, restricted movement, and impaired function [6]. From a biological perspective, the post-injury environment is typically marked by elevated levels of pro-inflammatory cytokines, catabolic enzymes, and immune cells. Furthermore, degenerative changes linked to OA can initiate a feedback loop that further diminishes the meniscus’s ability to heal effectively [7].

While arthroscopic diagnostic arthroscopy have been described as the gold standard for assessing meniscal injuries, diagnostic imaging is still necessary before any surgery. While various radiological tests have been described in the literature to assess these injuries, classical X-ray of the knee is rarely used and is not useful for the assessment of meniscal injuries. The gold standard for meniscal imaging is magnetic resonance imaging (MRI), due to its non-invasiveness and accuracy [1,8,9]. When examining treatment methods for these injuries, it is important to highlight the significance of the widely practiced meniscectomy [10]. Although initial treatment is often nonoperative, persistent and more complex meniscus tears generally require surgical intervention. Over the past two decades, the rate of meniscus repairs has risen significantly due to advancements in surgical techniques and improved postoperative outcomes. While recovery tends to be longer compared to meniscectomy, accelerated rehabilitation programs have shown promising results [11]. Sixty years ago, researchers discovered that removing the meniscus from the knee joint led to the degeneration of articular cartilage and the progressive onset of arthritis. Studies highlighted the anatomical and functional importance of the knee meniscus, sparking extensive research into diverse treatment strategies [12]. The primary method most commonly used for treating meniscal abnormalities was partial or total meniscectomy [13].

By 1982, partial meniscectomy emerged as a proposed alternative to complete meniscectomy. This finding marked a significant shift in the management of meniscus-related issues [12]. However, is partial meniscectomy the only method of treating meniscus damage?

This review explores the current understanding of the anatomical and biomechanical properties of the knee meniscus. This review focuses on the afore-mentioned meniscus injuries, with particular attention given to the diagnostics and methods used for their treatment. Lastly, an outlook on the future repair techniques of meniscus is provided.

## 2. Anatomy

The medial and lateral menisci are smooth and slippery, crescent-shaped structures in the knee joint. They are located on the medial and lateral aspects of the knee [1,14]. The menisci provide an effective joint connection, between the concave femoral condyles and the relatively flat tibial plateau [1]. The menisci are roughly triangular in a cross section with a concave superior surface to accommodate the femoral condyle’s convex surface and flat inferior surface to match the relatively flat tibial plateau [1,14,15]. Both the medial and lateral menisci are secured to the outer edges by coronary ligaments, also known as the meniscotibial ligaments [14]. When extracting two ends of the meniscus, each of them has an anterior and posterior horn [16].

**Figure 1:**
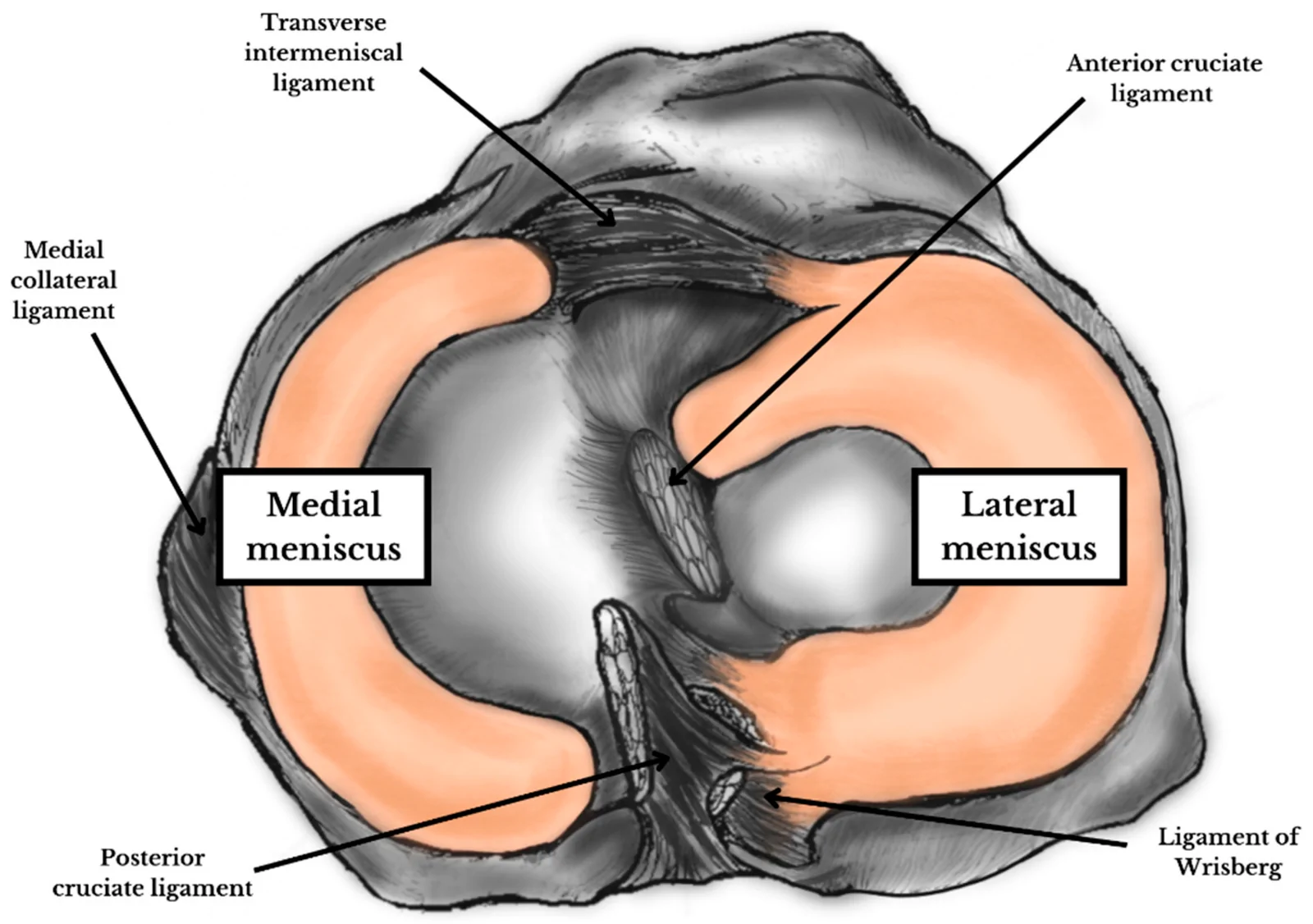
The anatomy of the meniscus viewed from above.

The menisci are connected to the tibia, femur, and each other through ligaments. The main ligaments that attach the menisci to the tibia are medial collateral ligaments; those that attach them to the femur are anterior meniscofemoral ligaments, also known as ligaments of Humphry, posterior meniscofemoral ligaments, also known as ligaments of Wrisberg, and medial collateral ligaments; and those that attach the menisci to each other are anterior or transverse intermeniscal ligaments [17]. There are two more important ligaments in the knee joint connecting the femur to the tibia: the anterior cruciate ligament (ACL) and the posterior cruciate ligament (PCL) (Figure 1).

### 2.1. Medial Meniscus

**Figure 2:**
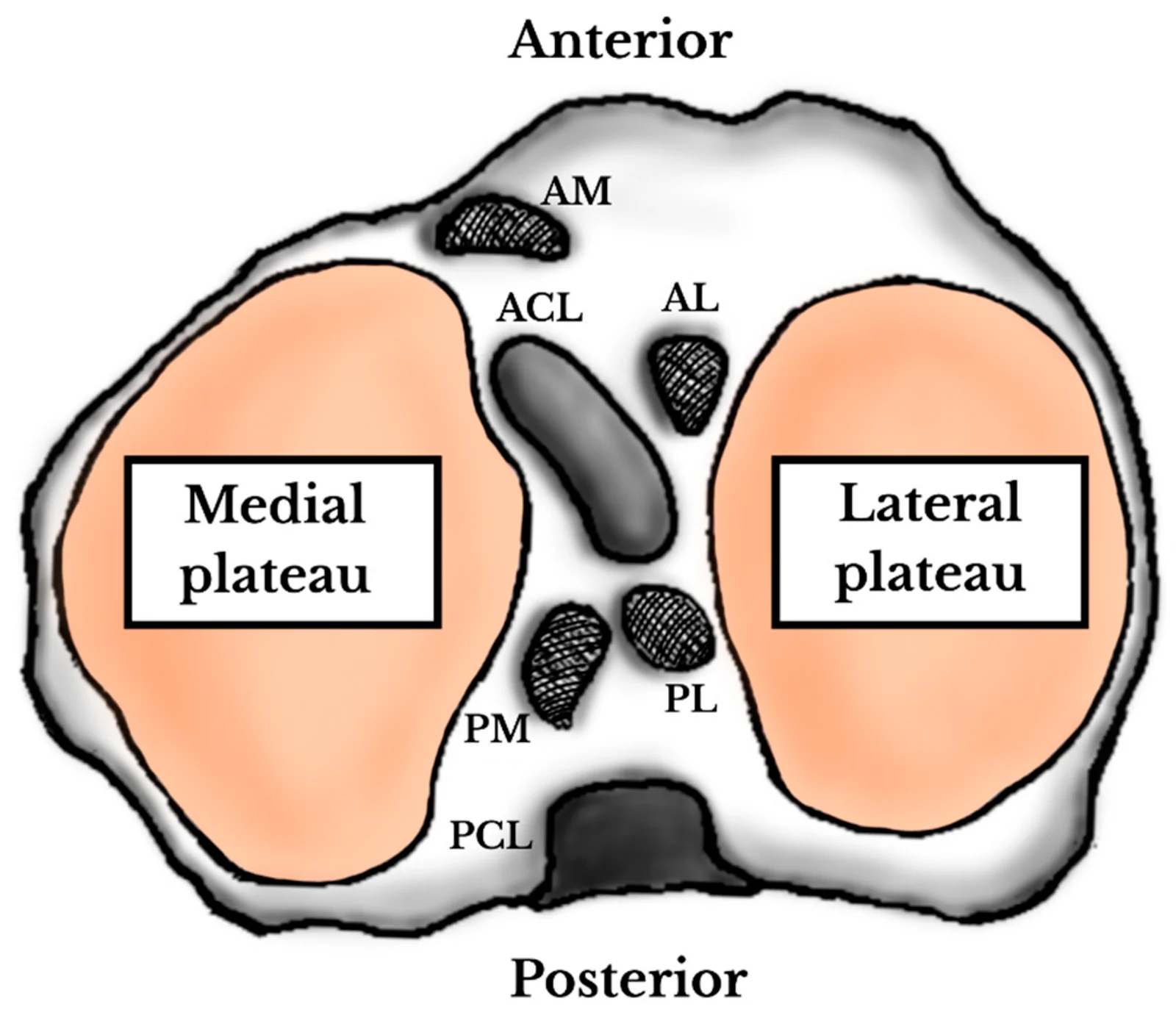
Meniscus horn insertion sites viewed from above. AL: anterior horn lateral meniscus; AM: anterior horn medial meniscus; ACL: anterior cruciate ligament; PCL: posterior cruciate ligament; PL: posterior horn lateral meniscus; PM: posterior horn medial meniscus.

The medial meniscus is C-shaped and covers between 51% and 74% of the medial tibial plateau surface area [1,15]. The narrower anterior horn is connected to the tibia by the anterior cruciate ligament (ACL) in the anterior intercondylar area. In the posterior intercondylar area, the wider posterior horn is positioned in front of the posterior cruciate ligament (PCL) and in the back of the posterior horn of the lateral meniscus (Figure 2) [15,18,19].

The ACL and PCL are two important ligaments in the knee joint connecting the femur to the tibia. They work in conjunction to keep the knee joint stable and prevent the tibia from moving too far forward relative to the femur [15,18,19].

### 2.2. Lateral Meniscus

The lateral meniscus is nearly circular in shape and covers between 75% and 93% of the lateral tibial plateau surface area [14,15]. The anterior horn of the lateral meniscus is only connected to the intercondylar fossa near the wide attachment area of the ACL and is linked to the anterior horn of the medial meniscus via the transverse ligament. The posterior horn of the lateral menisci is positioned anteriorly from the posterior horn of the medial meniscus, and it is linked to the inner part of medial femoral condyle via the posterior meniscofemoral ligament (ligament of Wrisberg) and the anterior meniscofemoral ligament (ligament of Humphrey). They are crucial in providing mobility and stabilization. Typically, one of them is present, and the Humphrey ligament is more common. Additionally, it is attached to the popliteus tendon [1,20,21,22]. The lateral meniscus is more mobile and is not anchored to the lateral collateral ligament. The attachment of the lateral meniscus to the femur and the popliteal tendon links its movement with that of the femoral condyle during rotation. As a result, it is less prone to injury compared to the more stationary medial meniscus [23].

## 3. Microstructure

The meniscus is composed of a dense extracellular matrix mainly consisting of water (65% to 72%) and collagen (20% to 25%), interposed with local cells and other components, which are glycosaminoglycans (17%), DNA (2%), adhesion glycoproteins (<1%), and elastin (<1%) [1,14]. Collagens are the key to providing tensile strength in the meniscus, making up about 75% of the extracellular matrix’s dry weight. In the red-red zone, type I collagen is the most abundant, comprising 80% of the dry weight, followed by other collagen types, such as II, III, IV, VI, and XVIII, but they are present only in <1% [1,12,14]. In the meniscus superficial layer, type I collagen fibers are oriented in a radial direction. In contrast, in the deep layer, collagen fibers are oriented circumferentially, with occasional radial “tie” fibers in the deep zone that interlace with the circumferential fibers to prevent longitudinal splitting. The surface layer contains randomly oriented fibers, which help reduce friction and create a smooth surface [1,14,15]. In the white-white zone, there are only two types: type II collagen (60%) and type I collagen (40%), which make up 70% of the dry weight [14,16,24].

Proteoglycans make up less than 1–2% of the extracellular matrix but play a crucial role. These large, negatively charged, and hydrophilic molecules draw in water, aiding fluid movement and helping to minimize compressive strain and potential injury to the meniscus [15]. As one of the main components of mature and immature fibers, elastin is present in less than 0.6% [12].

Pasiński et al. noted that the organic dry mass, 8–13%, consists of non-collagenous matrix proteins, including fibronectin. They are engaged in many cellular processes such as tissue healing, embryogenesis, coagulation process, cell migration, and adhesion. Other proteins, such as 2–6 kDa cationic peptides, known as β-defensins, which are connected to proteoglycans, aid in the protective function of meniscal tissues, such as anti-bacterial, anti–fungal, and anti-viral actions [16].

### 3.1. Meniscus Cells

The characterization of meniscus cells in contemporary literature remains somewhat contentious, with various terms being used, such as fibrocytes, fibroblasts, meniscus cells, fibrochondrocytes, and chondrocytes [12,25]. In the more superficial layer, cells have an oval, fusiform shape and are similar in appearance and behavior to fibroblasts [26].

These cells also possess long extensions, enabling communication with other cells and interaction with the extracellular matrix. This makes the outer portion of the meniscus akin to fibrocartilage [27].

In contrast, in the deeper part of meniscus, more rounded cells are embedded in an extracellular matrix, primarily composed of type II collagen, with a substantial proportion of type I collagen and a higher concentration of glycosaminoglycans. This gives the area a greater resemblance to hyaline articular cartilage. Consequently, the cells in this region are classified as fibrochondrocytes (chondrocyte-like cells). A third cell population has also been identified in the superficial zone of the meniscus. These cells exhibit a flattened, fusiform morphology and lack cell extensions. It has been proposed that they may represent specialized progenitor cells with potential therapeutic and regenerative properties [12,26,28].

### 3.2. Vascularization

**Figure 3:**
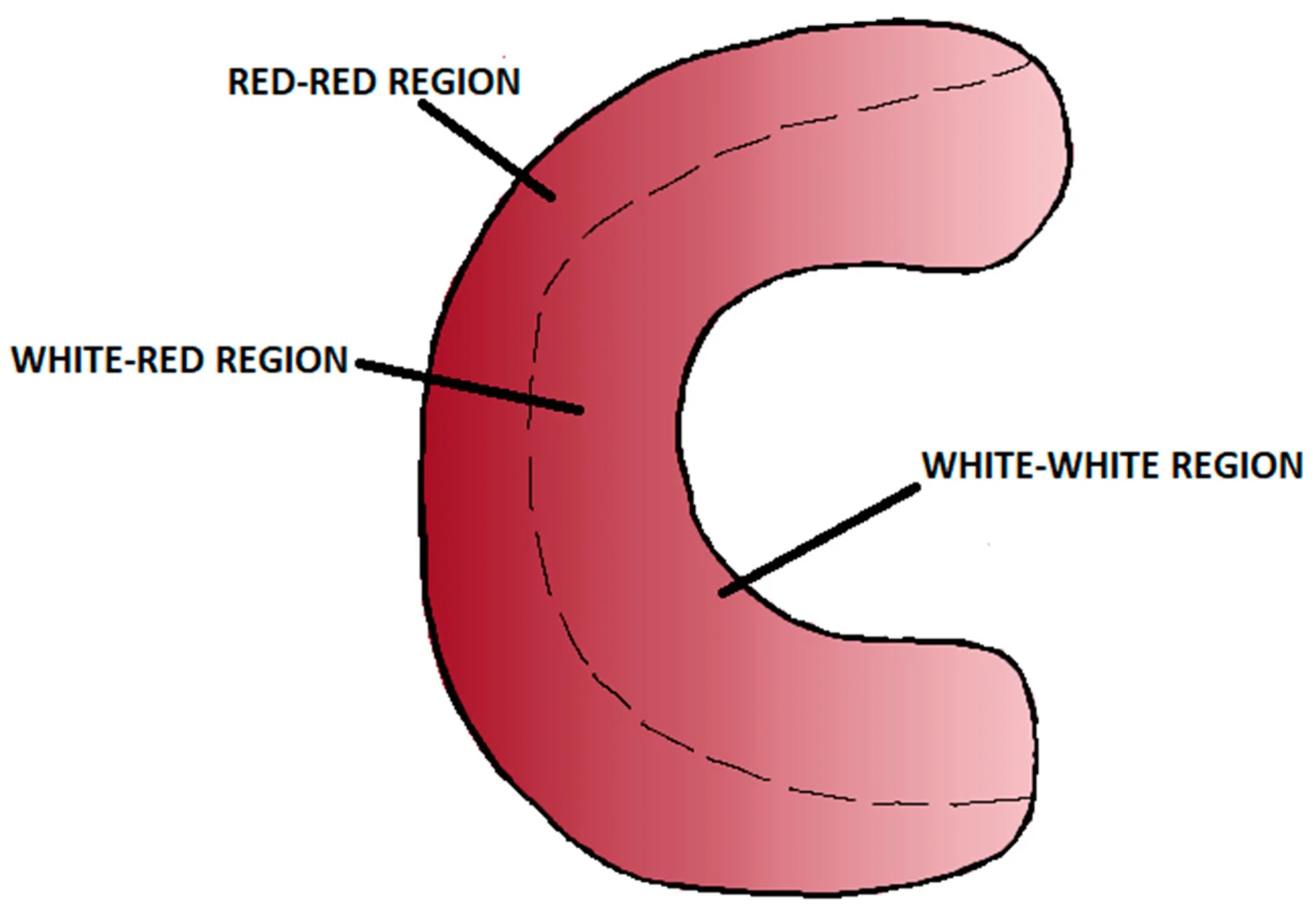
Anatomical variation in meniscal vascularization.

According to vascularization, we can distinguish three areas with different vascularization pattern. The red-red zone is a highly vascular region, and the white-white zone is a avascular and aneural region. Between these, the red-white zone is localized, and it has mixed properties of either the red-red or white-white zone (Figure 3) [12].

The medial and lateral middle geniculate arteries are branches of the popliteal artery and are responsible for providing blood supply to the meniscus [29]. These branches constitute a surrounding perimeniscal capillary plexus that penetrates to a depth of 2–3 mm within the menisci [15]. This leads to the belief that vascularization is limited to the peripheral 10–25% red-red zone of the lateral meniscus and 10–30% red-red zone of the medial meniscus, which has important implications for healing [1,29]. The remaining one-third of the meniscus, the white-white zone, obtains nutrients through synovial diffusion, while the transitional red-white zone in the middle has attributes of each zone [1,30].

The vascularization pattern and nutritional status of the menisci have very important implications for the potential healing and success of meniscus surgery [31]. The healing potential of a meniscal tear is largely dictated by the tear location [20]. Tears in the red-red and, to a lesser extent, red-white zones are considered to have a good healing potential, making repair commonly recommended. In contrast, tears in the white-white zone have been believed to have a poor healing potential [15]. The study by Chahla et al. identified mesenchymal progenitor cells in all three zones of the meniscus, including the white-white zone. This finding suggests that even the inner region of the meniscus, previously believed to lack regenerative capacity, may have some potential for repair. These cells can differentiate into bone, adipose, and cartilage tissue, underscoring their crucial role in the regeneration process [32].

Additionally, advanced imaging techniques, such as three-dimensional immunofluorescence and the uDisco method, revealed the presence of blood vessels in the white-white zone, particularly along its periphery. This discovery challenges the long-standing belief that this area is entirely avascular and incapable of self-regeneration [32].

Historically, meniscectomy was the preferred treatment for injuries in this zone. However, recent studies suggest that biological approaches, such as progenitor cell mobilization and growth factor stimulation, can significantly improve treatment outcomes. Furthermore, research has demonstrated that meniscal repair, even in areas with limited vascularization, can yield favorable clinical results, representing a major advancement in the treatment of meniscal injuries [32]. Additionally, Cinque et al. observed that meniscal repair significantly improved patients’ symptoms at 2 years follow-up regardless of the tear zone [33].

Meniscus vascularization plays a crucial role in its regenerative capacity. One strategy used to enhance healing involves improving blood supply by creating vascular access channels and tunnels, facilitating blood flow and the delivery of mesenchymal stem cells (MSCs) to the injured area [12].

Alternatively, vascularized synovial flaps and fibrin clots can support regeneration by providing growth factors and reparative cells. While these methods show promise, further research is needed to confirm their effectiveness [12].

### 3.3. Neuroanatomy

Menisci receive their nerve supply from the posterior articular branch of the posterior tibial nerve, terminal branches of the femoral, obturator nerves, and the common peroneal nerve. All of these penetrate the capsule and follow the same distribution as the vascular supply [14,29]. Like the vascular supply, innervations are most concentrated in the red-red zone of the menisci, with no neural elements in the white-white zone. Additionally, the anterior and posterior roots have more innervations compared to the meniscal body [14].

There are three types of mechanoreceptors present in the meniscus: Ruffini endings (type I), Pacinian corpuscles (type II) and Golgi tendon organs (type III). Pacinian corpuscles and Golgi tendon organs are more common than Ruffini endings [34]. Mechanoreceptors contribute to joint proprioception and afferent sensory input. Ruffini endings are unmyelinated, slowly adapting sensory fibers that detect changes in joint deformation and pain. Pacinian corpuscles are myelinated, rapidly adapting sensory fibers that respond to changes in pressure and tension. Golgi tendon organs, also myelinated and rapidly adapting, play a role in neuromuscular inhibition at terminal ranges of motion [14,29,34].

## 4. Biomechanics

The main roles of the meniscus include vital functions such as improving joint congruity and stabilization, load transmission, and shock absorption; increasing joint contact area; and decreasing joint contact stresses, joint nutrition, and lubrication (Table 1) [29,35].

### 4.1. Load Transmission

Around 40–60% of the load on an extended knee joint is transmitted to the meniscus, with 65–70% on the lateral side and 40–50% on the medial side. During the flexion motion, this load distribution increases up to 90% [1]. Load bearing is directly related to applied compressive forces of the knee, generating contact stresses. Thus, a larger contact area over which the load is distributed reduces the contact stresses on that area [1,17]. The femoral condyle and the tibial plateau are both convex and on the lateral side of the knee, which results in a mismatch between them. In contrast, the medial femoral condyle articulates with a concave medial tibial plateau; thus, we see a better congruity [17,29].

### 4.2. Shock Absorption

The shock-absorbing function of the menisci is linked to their viscoelastic properties, primarily due to the fact that the extracellular matrix of the meniscus is mainly composed of water. The fluid drag forces absorb the shock from external forces [1]. During the motion, the vibrations are transmitted through the knee and are partially absorbed by the menisci [14]. Voloshin et al. observed that excision of the meniscus reduces the knee’s shock absorbing capacity by 20% [24]. This demonstrates that, after meniscectomy, the shock absorption capacity is reduced, increasing the risk of osteoarthritis (OA) [14].

### 4.3. Stability

The lateral meniscus has greater mobility compared to the medial meniscus, enabling it to move along with the lateral femoral condyle, which experiences more forward–backward movement because of the larger curvature difference between the lateral femoral condyle and the lateral tibial plateau. In contrast, the medial meniscus is more restricted in movement on the medial side and acts as a secondary stabilizer [14,29]. The intact meniscus restricts excessive movement in all directions, aiding in knee joint stability. This stability is further supported by the soft tissue structures of the knee joint capsule [1].

### 4.4. Joint Lubrication and Nutrition

The crucial role of the menisci in joint lubrication is known, but the mechanism is not yet fully understood. Lubrication is facilitated by synovial fluid and specific proteoglycans. The viscous synovial fluid, produced by synoviocytes, is believed to reduce shear forces between the meniscus and articular cartilage during joint movement and weight-bearing activities. The hydrophilic mucin-like end of proteoglycan, known as lubricin, participates in this process. Together, they lower the static and dynamic friction coefficient [1,14,29].

## 5. Injuries

Knee cartilage volume loss often results from meniscal damage or meniscal excision. Meniscal tears easily lead to osteoarthritis and are inherently associated with osteoarthritis. Nowadays, it is known that modern treatment methods for saving meniscal tissue have an advantage over meniscectomy [5]. Knee osteoarthritis is known as common degenerative and chronic joint disease, resulting in daily pain, swelling, and stiffness of the joint [36]. Meniscus tears are among the most frequent injuries managed by orthopedic surgeons. In the past, meniscectomy was commonly performed, but now, biomechanical and clinical studies have shown the crucial role of the meniscus in impact absorption and force distribution within the knee [37]. There are two groups of meniscal injuries: traumatic and degenerative. The first group includes meniscus tears, which result from sudden, traumatic knee injury. The second group includes degenerative meniscus lesions, which arise from fibrocartilage progressive degeneration [38].

**Figure 4:**
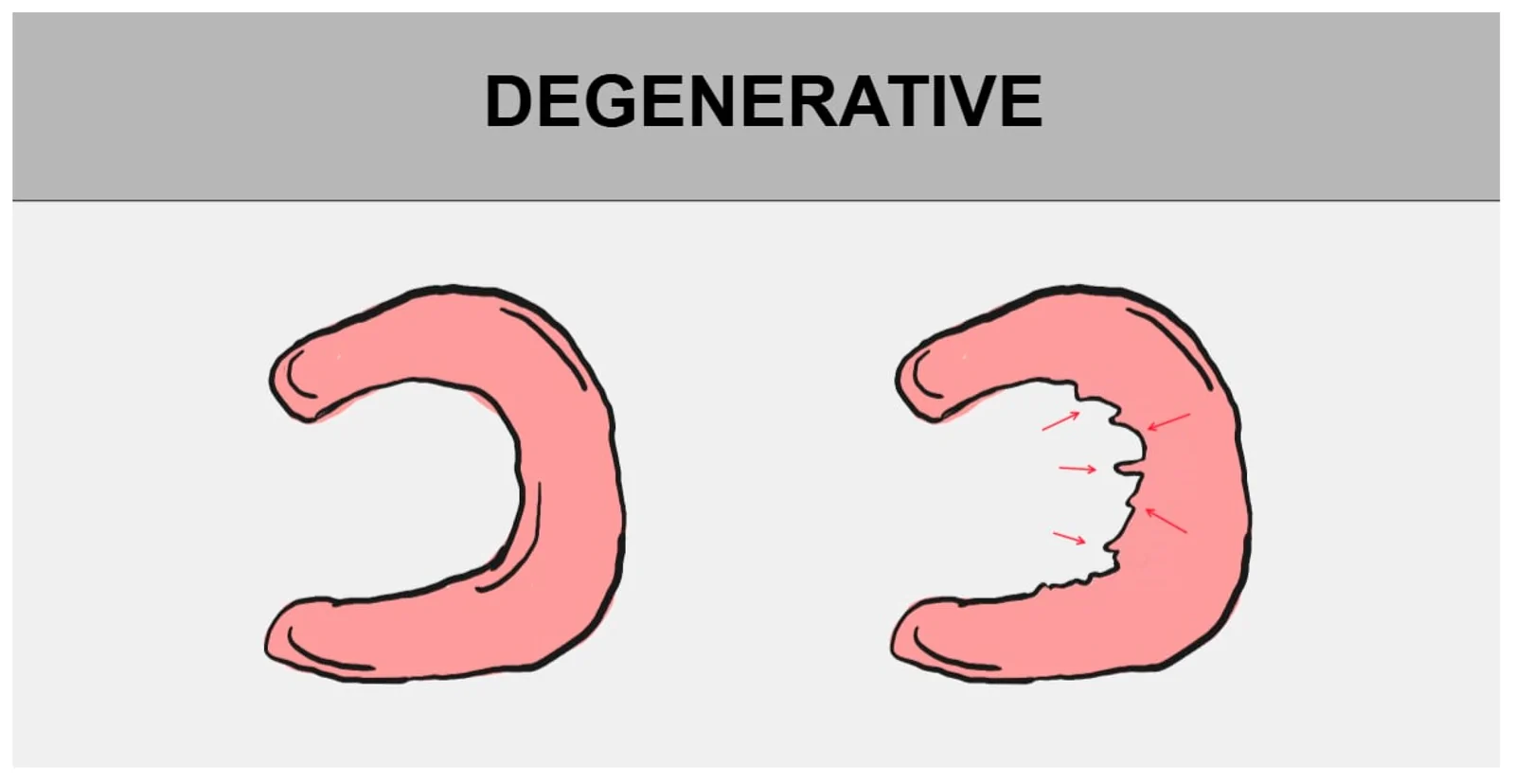
The anatomical structure of the meniscus and a degenerative tear.

The division of meniscal tears is based on their tear pattern. There are several types of them, such as longitudinal tear, radial (transverse) tear, and horizontal (cleavage) tear. They may progress to bucket handle tear, parrot break tear, and flap tear, successively [1,12,29]. All of the above-mentioned damages may be consequences of degenerative meniscus tears, a normal part of aging (Figure 4) [39].

Terzidis et al. showed that longitudinal, bucket handle, and oblique tears are the most frequent on the medial side, whereas radial and horizontal tears are the most frequent on the lateral side [8]. Determining which lesion you are dealing with is extremely important when choosing the most appropriate therapy.

### 5.1. Bucket Handle Tear

**Figure 5:**
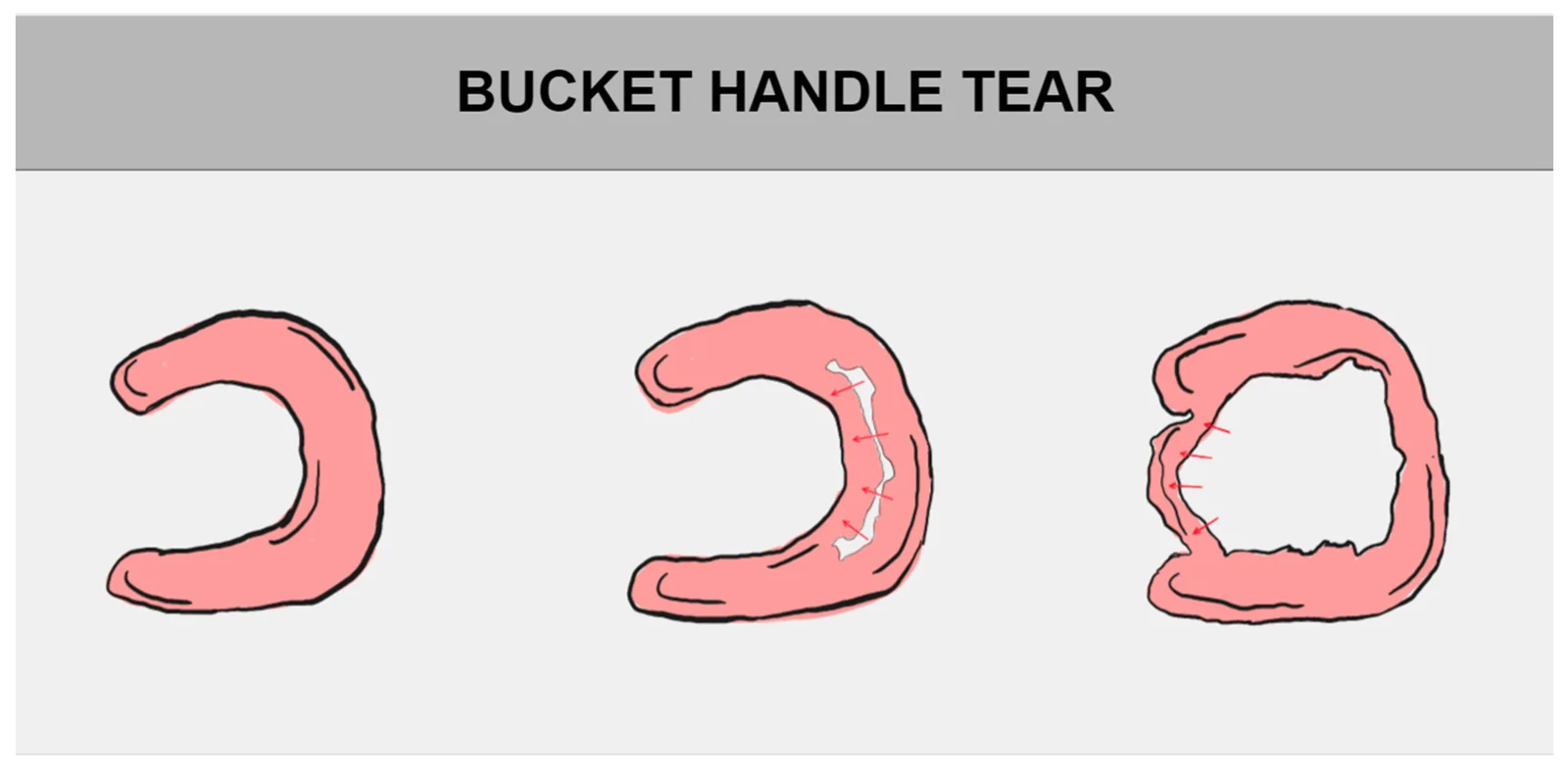
The anatomical structure of the meniscus and progressive damage from a longitudinal tear to a bucket handle tear.

One of the most frequent meniscal tears are bucket handle tears, usually found in the medial meniscus (Figure 5) [40].

The longitudinal tear consists of two fragments, the inner handle and donor bucket. A displaced handle is attached to the body of the meniscus by both ends [41]. Bucket handle tears are an exceptional challenge due to their degree of advancement and frequency. Very often, the result of this type of damage is a characteristic mechanical block, represented by the patients as knee locking, audible and palpable clunks, as well as functional impairment, often causing loss of knee extension [40,42,43]. Cerciello et al. observed that, while the diagnostics may be difficult and extreme, the “Rising Moon” sign (the elective pain at the anterior aspect of the joint line) has shown to be a useful tool in daily life practice with very high specificity and sensitivity [40].

In the treatment of bucket handle tears, both the arthroscopic inside-out and all-inside techniques are used. The inside-out technique prevents anatomical bucket handle repair mismatch [44]. Furthermore, partial meniscectomy is performed arthroscopically and is utilized in specific cases [45].

### 5.2. Parrot Beak Tear

**Figure 6:**
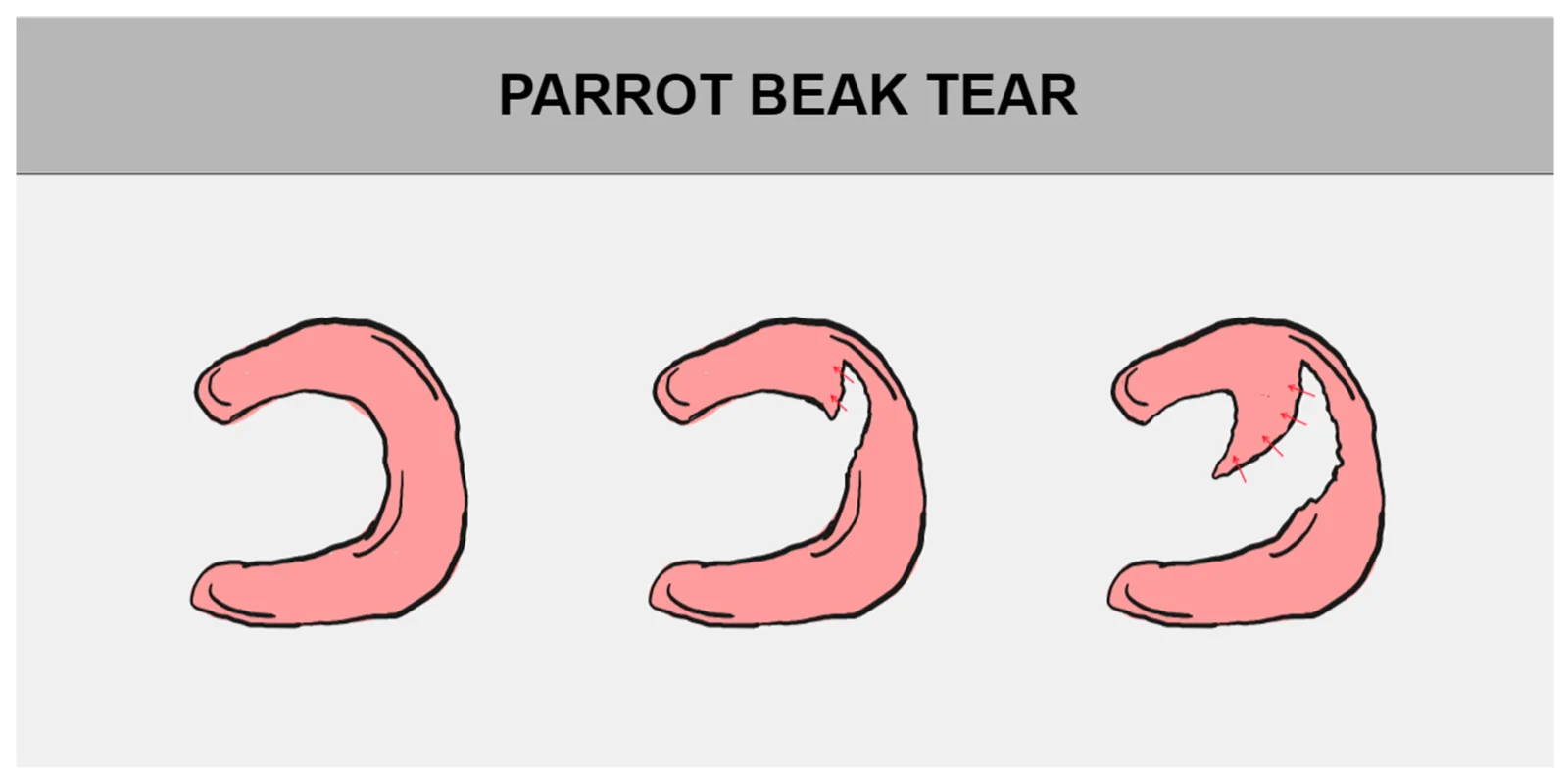
The anatomical structure of the meniscus and progressive damage from a radial tear to a parrot beak tear.

A parrot beak tear is typically encountered in isolated injuries in the avascular zone. The tear owes its name to the shape, which resembles a parrot’s beak. It is a characteristic feature of oblique or radial tears, where the free edge of the meniscal flap is displaced inward (Figure 6) [46,47].

The flap curves towards the meniscus, resembling the above-mentioned parrot’s beak. It is also referred to as the “meniscal comma sign” [46,48]. It is a complex injury that starts as a central radial tear and progressively extends toward the periphery, ultimately creating a detached fragment that can subsequently be displaced into the meniscal recess [48,49]. This type of damage has a negative impact on the load-bearing function of the knee joint, especially in the middle part of the lateral meniscus, due to the fact that the lateral meniscus is responsible for about 70% of the load transmission across the knee joint [47,50].

On the other hand, it also involves the medial meniscus, particularly in the body and posterior horn. In these cases, the detached fragment may migrate to the intercondylar notch or the meniscal recess. The displaced fragment is more likely to move to the meniscotibial recess than to the meniscofemoral recess, with downward displacement being more common than upward [49]. In the deep medial collateral ligament, tension is created and becomes the cause of significant pain [48].

Radial tears in patients with OA are typically treated with a partial meniscectomy. The management of radial tears is categorized into two types: partial tears (affecting the inner edge), which are treated with a partial meniscectomy, and complete tears, which are repaired using all-inside or inside-out horizontal mattress sutures [44]. In the treatment of radial tears, the outside-in suture technique is also used [51].

### 5.3. Horizontal Tear

**Figure 7:**
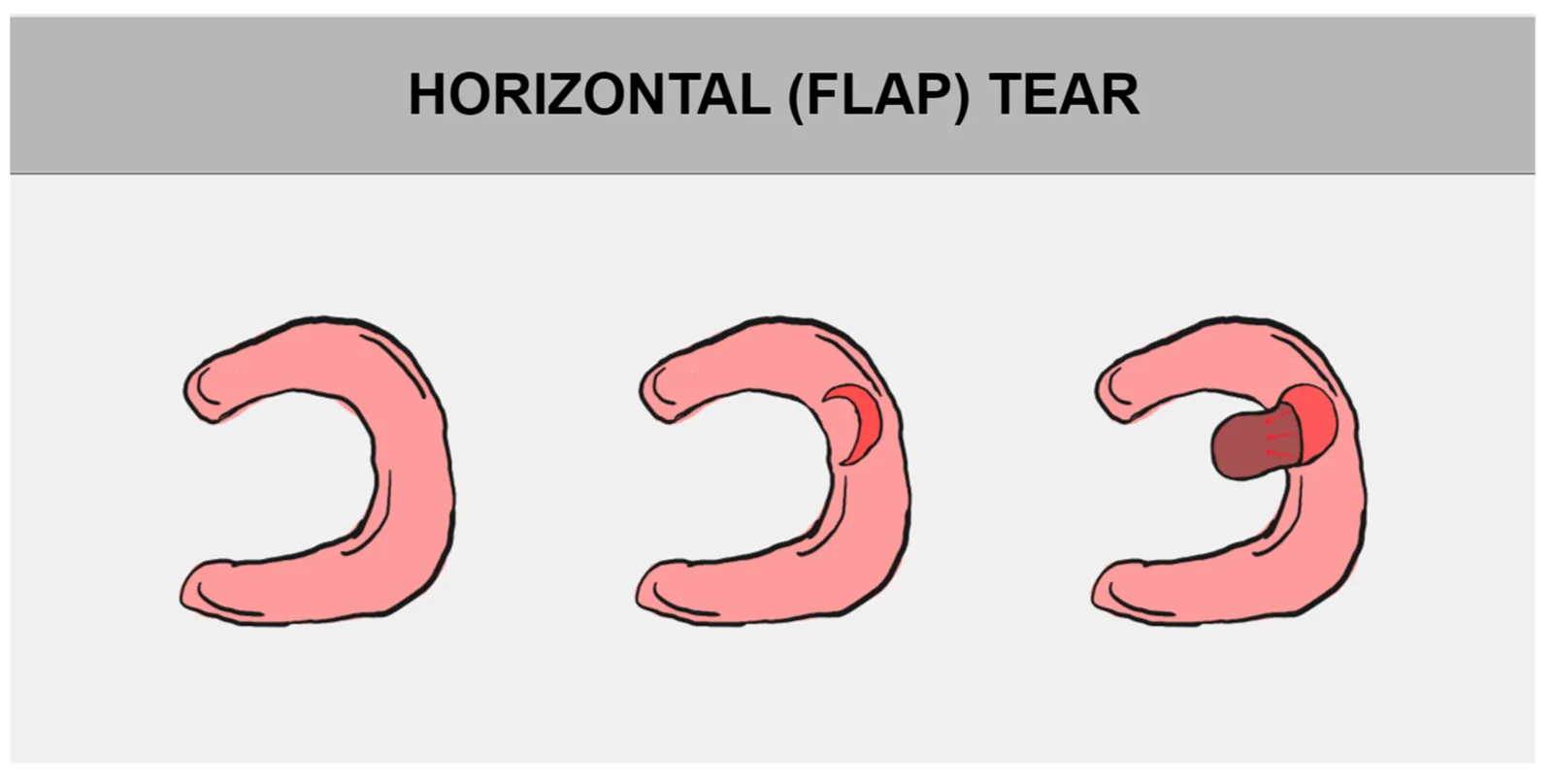
The anatomical structure of the meniscus and progressive damage from a horizontal tear to a flap tear.

Horizontal cleavage tears are complex meniscal injuries and are the most common type of tears. The meniscus is divided into superior and inferior leaflets, affecting the central, less vascular zones of the meniscus (Figure 7) [15,16].

The etiology of horizontal cleavage tears is not fully known. They can be either traumatic or degenerative, with traumatic ones more frequently occurring early in life [52]. Degenerative meniscal lesions progress slowly, often appearing in older age. They usually are linked to osteoarthritis and meniscal degeneration. The most common location is in the posterior portion of the medial meniscus [10,16]. In arthroscopic surgery for medial meniscus horizontal tears, unstable flaps are often found, which may be related to persistent pain [53]. Horizontal cleavage tears have been traditionally treated with nonsurgical methods or a partial meniscectomy. However, due to the unpleasant consequences of partial meniscectomies, the surgical repair of horizontal cleavage meniscal tears is increasingly performed in suitable patients. New treatment methods also provide better long-term outcomes and increase cost savings [13].

Non-surgical treatment includes medication, such as non-steroidal anti-inflammatory drugs and analgesics, along with recommended physical exercises to enhance muscle strength, and physiotherapy [54]. Surgical treatment involves arthroscopy, utilizing all-inside and inside-out sutures [13,55]. Meniscal repair techniques may also include a biologic component, such as fibrin clot insertion at the repair site, PRP application, or concurrent marrow stimulation to promote healing [44,56].

## 6. Diagnostic

### 6.1. Magnetic Resonance Imaging (MRI)

**Figure 8:**
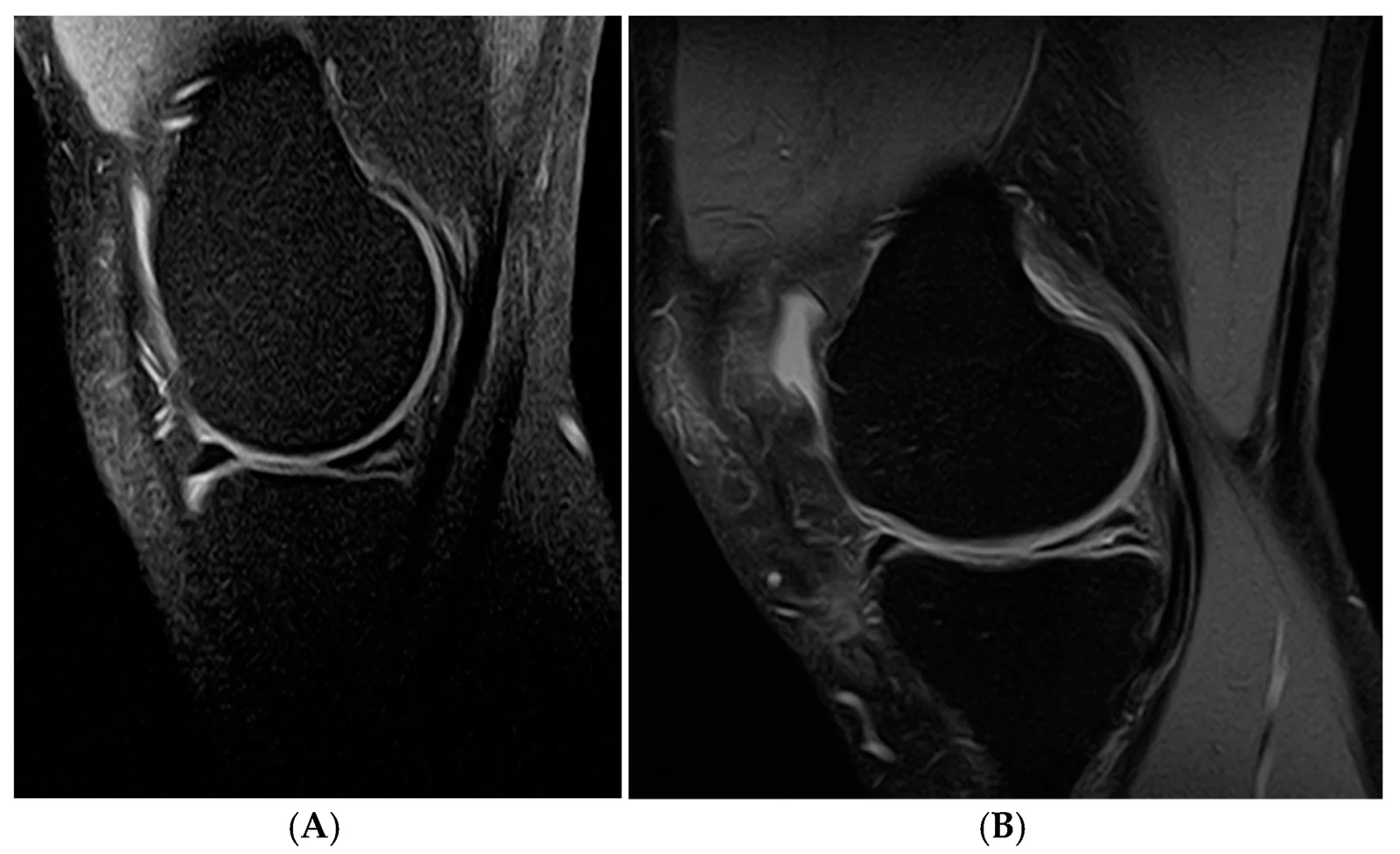
(**A**,**B**) MRI images of menisci lesions.

Magnetic resonance imaging (MRI) is one of the most accurate imaging tests that allows for a detailed assessment of the internal structures of the knee joint, such as ligaments, menisci, cartilage, and soft tissues. By using a strong magnetic field and radio waves, MRI provides precise images without the need for X-rays. Therefore, MRI provides the evaluation of the internal derangement of the knee [57]. It is important to highlight that MRI enables the analysis of meniscal abnormalities in all spatial planes with high resolution. Its multiparametric capabilities allow for the visualization of specific injuries or structures, depending on the selected sequences. This technique provides a comprehensive assessment of the stability of the tear and the risk of its progression. Additionally, MRI helps determine whether a meniscal tear is amenable to preoperative repair, which is a crucial factor in treatment planning. It helps to avoid unnecessary invasive diagnostics, such as knee arthroscopy [9]. It is a commonly used diagnostic method for meniscal pathology [49]. In the imaging diagnostics of meniscus lesions, MRI is known as the “gold standard” (Figure 8A,B).

Misinterpreting a parrot beak tear as a bulging meniscus with concomitant osteoarthritis on MRI often results in overlooking the true pathology, and this delays treatment [48]. The recent characterization of the parrot beak tear as the meniscal comma sign is associated with the displacement of the meniscal flap into the meniscotibial recess, which, on MRI, resembles an “meniscal apostrophe sign” [48,49]. This allows for the improved diagnosis and classification of this type of injury, leading to more appropriate surgical treatment.

In the case of bucket handle tears, MRI may reveal, among other findings, the absent bow-tie sign, the fragment-in-notch sign, the double posterior cruciate ligament sign, the truncated meniscus sign, the double anterior horn sign, and a flipped medial meniscus [42]. On the other hand, horizontal tears in the MRI are classified on a scale of 1 to 3, with grade 3 being the lesions that penetrate the outer fibril layers and open into the joint [52].

The diagnostic sensitivity for medial meniscus tears ranges from 86% to 96%, with specificity ranging from 84% to 94%. For lateral meniscus tears, sensitivity ranges from 68% to 86%, while specificity ranges from 92% to 98% [9]. Mackenzie R et al. reported that the overall sensitivity of MRI for detecting meniscal lesions was 88%, with a specificity of 94% [58].

The conclusion that can be drawn from this is that MRI is highly reliable for diagnosing medial meniscus tears, given its high sensitivity and specificity. However, the lower sensitivity for lateral meniscus tears suggests that MRI may occasionally miss these injuries, even though its specificity remains high. These findings highlight the fact that, while MRI is an excellent tool for diagnosing meniscal injuries, clinicians should be aware of its limitations, especially when it comes to detecting lateral meniscus tears.

The limited visualization of the lateral meniscus on MRI is attributed to its unique anatomy and location. As shown in the study by P. Krakowski et al., physical examination is more effective than MRI for assessing the lateral meniscus [59].

Additionally, P. Krakowski et al. found that physical examination was more accurate than MRI in diagnosing anterior cruciate ligament (ACL) tears. While MRI remains a valuable supplement to a comprehensive clinical evaluation, it should not replace a thorough physical examination [59].

MRI should primarily be prescribed to patients for the detailed assessment of injury morphology before surgery, rather than as a tool for confirming a diagnosis that can be accurately established through a comprehensive physical examination [59,60].

### 6.2. CT Arthrography

Arthrography is also a complementary diagnostic tool for meniscus tears, with an accuracy of up to 93% [9,61]. Arthrography is a diagnostic method that predates MRI. While MRI is now more commonly used due to its lack of ionizing radiation, arthrography can still be combined with computed tomography (CT) to create CT arthrography, which offers complementary information to MRI. CT arthrography is also a viable option for patients who are unable to undergo MRI due to factors such as orthodontic braces, pacemakers, metal plates, clips, or screws [9].

A complementary procedure to arthrography is also arthroscopy, and when used together, they increase the accuracy of diagnosis to 98.3%. This combination is used in more diagnostically challenging cases [62].

CT arthrography is also used to assess the postoperative meniscus, to evaluate cartilage and osteochondral damage, and to detect the presence of intra-articular foreign bodies [63].

### 6.3. Standard Radiography

Standard radiography is not useful for diagnosing or evaluating meniscal pathology, as the meniscus is not visible in this type of imaging. However, an X-ray is recommended to assess for possible coexisting osteoarthritis, which often develops in conjunction with meniscal damage. Additionally, radiographs can help exclude other conditions, such as osteochondritis or loose bodies within the joint, thanks to their ability to visualize the joint spaces [9].

### 6.4. Ultrasound

Similarly, ultrasound of the knee is commonly used as a diagnostic tool for tendon and ligament injuries, but it is rarely employed in the diagnosis and management of meniscal abnormalities [9]. De Flaviis et al. reported that ultrasonography using small-parts probes effectively detected minor structural irregularities, small meniscal cysts, and extra-articular effusion. This method is a reliable and classic approach for diagnosing degenerative meniscal cysts, which may result from other meniscal injuries [64].

According to the study by De Flaviis et al. [64] conducted on 27 patients, ultrasonography allowed for the classification of meniscal degeneration into three stages:

- Stage I (22% of patients): Minor surface irregularities, wall thickening, and a hypoechoic structure, with no knee swelling or abnormalities on X-ray.
- Stage II (30% of patients): Presence of microcysts (1–4 mm) accompanying structural changes.
- Stage III (30% of patients): Advanced necrosis leading to the formation of large cysts, sometimes with calcifications visible on X-ray.

## 7. Treatment Methods

### 7.1. Arthroscopy of the Knee

Minimally invasive surgery (MIS) has become the option of choice for both meniscal diagnosis and treatment. Banach et al. noted that reduced tissue damage, procedure and recovery time, and improved pain management and aesthetics are the key benefits of MIS [65].

Knee arthroscopy was initially introduced early this century but gained widespread acceptance and became a significant advancement in orthopedic surgery only within the past two decades. This technique has enabled the treatment of many disorders with significantly reduced patient morbidity [66]. Knee arthroscopy benefits from technological advancements that enhance the safety and success rates of procedures [65].

Providing spatial information about anatomical structures and surgical tools through visual navigation can significantly improve procedural safety and facilitate the integration of robotic assistance. Robotic surgical systems aim to enhance precision and maneuverability, minimize tremors, and offer 3D visualization alongside the active guidance of surgical instruments [49,65].

Wind et al. noted that numerous arthroscopic complications have been reported in the literature, including hemarthrosis, deep venous thrombosis and embolism, compartment syndrome, neurologic injury and reflex sympathetic dystrophy, popliteal artery injury, fluid dissection, fistula formation, pneumoscrotum, fat pad hemiation, breakage of instruments, and infection, but they also observed that most patients recover uneventfully following arthroscopic treatment for infected knee joints [67].

In a study conducted by Tsujii et al., patients underwent arthroscopic repair using the inside-out technique with reduction sutures. They observed that this technique obtained satisfactory results in patients with tears extending to the vascular zone, whereas those with tears in the avascular zone failed [50].

Training in arthroscopic techniques must be enhanced, and the understanding of knee conditions must be deepened to minimize potential complications and errors during arthroscopic procedures. Additionally, the misuse and overuse of knee arthroscopy must be addressed. To tackle this issue, implementing stricter audits that require photographic documentation of lesions both before and after treatment is recommended [66].

### 7.2. Traditional Meniscectomy

In the past, open total meniscectomy was regarded as the gold standard for managing meniscal tears due to the fact that menisci were considered to be functionless [5,68].

Traditionally, the indications for repair have focused on younger patients, traumatic injuries, and tear types that demonstrate the greatest healing potential, particularly longitudinal vertical tears located in the vascular zone [13]. Over time, partial meniscectomy and meniscal repair techniques have been developed. Meniscal repairs have progressed from open procedures to all-inside arthroscopic techniques [69], but Beaufils et al. observed that arthroscopic meniscectomy is still one of the most common procedures [10].

### 7.3. Present Meniscectomy

Meniscectomy is performed during a minimally invasive knee arthroscopy procedure. During the operation, the orthopedist performs meniscus debridement. The rule of thumb is to remove the smallest necessary part of the meniscus without unreasonable excess. Debridement may also include other structures of the knee, the removal of which is indicated to improve the quality of movement and reduce pain [70]. Meniscectomy can be carried out either completely or partially, using open surgery or arthroscopic techniques. The first arthroscopic meniscectomy is attributed to Masaki Watanabe, often regarded as the “father” of arthroscopy [71].

Meniscectomy continues to be one of the most commonly performed orthopedic surgeries. This high frequency remains concerning, given substantial scientific evidence supporting meniscal repair or nonoperative treatment for traumatic tears and similar approaches for degenerative meniscal injuries [10].

For example, parrot beak tears often cause the meniscal flap to become trapped in the joint during knee flexion. Due to their location in the avascular zone, these tears result in mechanical instability of the meniscus and have a limited capacity for healing. As a result, resection—specifically partial meniscectomy—is required to prevent further damage [47].

Traumatic tears, particularly longitudinal vertical tears in well-vascularized areas, have shown high success rates with repair, leading to faster recovery, improved function, and better cartilage protection. Asymptomatic lateral meniscal tears found during ACL reconstruction may not require intervention, while posterior ramp lesions, traumatic horn tears, and radial tears are often excellent candidates for repair. However, since the natural history of these injuries is not fully understood, nonoperative treatment may also be a valid consideration in certain cases [10].

All things considered, historically, meniscectomy was the go-to treatment, but recent randomized studies have demonstrated that arthroscopy offers no significant advantage over nonoperative management. Consequently, nonoperative care should be the first choice, with meniscectomy reserved for persistent symptoms or cases of severe mechanical issues. In younger athletes, horizontal cleavage tears require particular attention, as meniscal repair is essential to prevent the need for extensive meniscectomy, which could have significant consequences for their active lifestyles [10].

### 7.4. The Suturing Technique

One of the methods of treatment is the suturing technique. The inside-out technique remains the most commonly used method, where sutures are passed through the meniscus using a curved cannula and then tied over the joint capsule. The inside-out technique is particularly beneficial for bucket handle tears. It allows access to the middle third and most of the anterior third of the meniscus [72]. For example, due to the location of the parrot beak tear in the white-white zone, partial meniscectomy is recommended, as well as the innovative “inside out” method, including additional reduction sutures [47]. The outside-in technique can be helpful for addressing tears in the anterior third of the meniscus that are difficult to reach [72].

Each of the suturing techniques has its advantages (Table 2). The inside-out approach is applicable to all types of tears, including complex and hard-to-reach ones. Sutures are placed using smaller diameter, solid needles, which help maintain the structural integrity of the meniscus and reduce the risk of tear propagation. The inside-out suturing technique also reduces the risk of implant migration within the joint, minimizing the potential for injury to the articular cartilage surfaces. Furthermore, the inside-out technique offers cost savings, as sutures are more affordable than newer all-inside devices [55]. The outside-in technique is less commonly used and primarily reserved for mid-body or anterior third meniscal tears. However, it remains a favorable option due to its small incisions, low risk of neurovascular injury, and high success rates [73]. All-inside meniscus repair is becoming increasingly popular for treating vertical longitudinal and bucket handle tears, though it can also be used for a variety of tear patterns. This technique has been shown to reduce operative time and facilitate faster post-operative recovery [74]. Additionally, all-inside repairs are less technically demanding and minimally invasive, with a lower risk of injury to posterior neurovascular structures [73].

There are also disadvantages of using suturing techniques in meniscus treatment. The inside-out approach is often more technically demanding and time-consuming, frequently requiring a surgical assistant for needle retrieval. When using this technique, extra caution is needed to minimize the risk of injury to neurovascular structures, such as the saphenous and peroneal nerves. While retrieving the sutures, a broad retractor should be applied to shield the popliteal artery and its branches from potential damage by the exiting needles [55]. The outside-in suturing technique cannot be applied to all meniscus tear patterns and may cause wound problems [51]. The use of older, more rigid all-inside implants has been linked to articular cartilage injury, device migration, and device failure. Even newer all-inside devices can cause local soft tissue irritation and swelling [73]. Additionally, this treatment method carries the risk of the formation of meniscal cysts [75].

### 7.5. Repair Using All-Inside Compression Sutures

All-inside meniscal repair has become a widely used technique today, with its ease of use contributing to its popularity [76]. All-inside repair with only sutures using these suture passers is known as all-inside suture (AIS) repair, to set it apart from standard all-inside repair with implants or anchors. This AIS repair method enables suturing directly from meniscus to meniscus across the tear without the interference of soft tissues, such as the capsule, between the suture and meniscus, which allows the torn edges to close directly. For this reason, AIS repair is considered an anatomical method of meniscus repair [69]. All-inside fixation devices are especially useful for tears in the posterior third, as they reduce the risk of neurovascular damage caused by outside-in needles. These devices can also be used for middle third tears [72].

The alternative technique involves using an autologous strip of the quadricep tendon to bridge the gap between the upper and lower meniscus leaflets, followed by the application of an all-inside compression suture. This surgical technique is used to repair a horizontal cleavage tear in the posterior horn of the medial meniscus in adolescent or young adult patients [52].

In standard diagnostic arthroscopy, a probe is utilized to assess both menisci. In this method, a graft is harvested from the quadricep tendon, measuring 10 mm in width and 20 mm in length. It is then prepared by placing sutures, two non-absorbable sutures and peripheral absorbable compression sutures. The graft with sutures is then used to bring together and compress the superior and inferior leaflets of the torn meniscus. This treatment method aims to preserve the meniscal anatomy and volume, while also sealing the meniscus to prevent the development or recurrence of a parameniscal cyst [77].

There are also other methods of treating horizontal meniscal tears that apply all-inside circular compression sutures. One of these utilizes the biological augmentation of meniscal repairs with a marrow venting procedure. This procedure is usually performed on young patients who have not developed degenerative changes yet. Access to the knee joint is achieved arthroscopically using a standard approach. An arthroscopic probe helps assess the tear’s shape, size, and stability. The repair is performed by applying the self-passing, all-inside Novostitch Pro device (Smith & Nephew, Andover, MA, USA). Circumferential compression stitches enable precise anatomical alignment and effective compression of the meniscal leaflets. After completing the meniscus repair, four to five holes may be made in the lateral aspect of the intercondylar notch using a microfracture awl. This allows bone marrow elements to flow into the joint space. The marrow venting procedure creates a biologically enriched environment that supports meniscal healing by releasing peptides, growth factors, and pluripotent cells from the bone marrow. Compared to isolated meniscal repairs, the marrow venting procedure has demonstrated improved clinical outcomes. This technique is also relatively simple and utilizes a self-passing suture device, enabling secure suture placement while protecting neurovascular structures [52]. Studies have shown that both inside-out and all-inside techniques have similar healing rates, functional outcomes, and complication rates [72].

**Table 2:** Table shows indications, complications, and success rates of meniscus suturing techniques.

Meniscus Suturing Technique	Indications	Complications	Success Rate
Inside-out	Bucket handle tear [78]	Postoperative stiffness [78] Injuries of saphenous and peroneal nerve [75]	80–100% [78]
Outside-in	Radial tear [51]	Wound issues [51] Neurovascular damage [72]	86% [79]
All-inside	Bucket handle tear [78] Horizontal tear [13]	Postoperative stiffness [78] Injuries of neurovascular structures [75] Meniscal cyst formation [75]	79.5–93.1% [78] 77.8% [13]

### 7.6. Platelet-Rich Plasma

Platelet-rich plasma (PRP) is a biological product derived from the plasma portion of autologous blood, characterized by a platelet concentration higher than the baseline level. Its use is becoming increasingly popular across various medical fields, particularly in orthopedics [80]. PRP therapy promotes meniscal healing by delivering growth factors and anti-inflammatory agents. It serves as an excellent alternative to failed treatments or as a supplementary option for short-term management. Studies with follow-up periods of less than one year have demonstrated that platelet-rich plasma injections for meniscus injuries significantly improve knee symptoms, including pain reduction and enhanced daily activity. Additionally, this therapy may help athletes with meniscal tears return to sports more quickly and requires a shorter rehabilitation period [81]. This treatment approach is also effective in enhancing clinical outcomes for adolescent patients with meniscus tears [56].

However, there are very little data in the literature assessing the effect of blood-derived products on the healing of chronic meniscal tears. Kaminski et al. conducted a randomized controlled trial, in which the control group of 30 participants underwent percutaneous meniscal trephination with placebo augmentation, while the PRP-treated group of 42 participants received percutaneous meniscal trephination with PRP augmentation. Augmenting this technique with PRP significantly enhances the rate of meniscal healing. Notably, this simple procedure may also reduce the need for future arthroscopic intervention. Findings from this study suggest that PRP augmentation offers substantial and clinically meaningful benefits [82].

In another randomized controlled trial, Kaminski et al. demonstrated that PRP increased the likelihood of meniscus healing by more than six-fold. Furthermore, the risk of adverse events associated with PRP use remains low [83].

In a randomized controlled trial, Alkhuzai et al. compared the effectiveness of orthobiological ozonized PRP and hyaluronic acid therapy following arthroscopic suturing and arthroscopic partial meniscectomy for the treatment of meniscal tears in degenerative knee osteoarthritis. The study included patients with pure knee joint OA degenerative type grades II and III, along with various types of meniscal tears, as confirmed by MRI of the knee joint. The study found that intra-articular orthobiologic treatments using ozonized PRP and hyaluronate were more effective than arthroscopic partial meniscectomy (APM) for managing degenerative knee osteoarthritis. Ozonized PRP, in particular, appears to be a safe option, offering notable reductions in inflammation, effective pain relief, and strong improvements in functional outcomes [84]. Despite promising research results, the long-term effectiveness of PRP remains uncertain; thus, further, in-depth research is necessary.

### 7.7. Synthetic Implants

One of the treatment options for meniscal replacement is allograft implantation. However, its application is limited by challenges such as availability, size compatibility, and cost. In this context, synthetic meniscus implants offer significant potential advantages [85]. Currently, several synthetic meniscus implants have been in use, such as the collagen meniscus implant (CMI, Ivy Sports Medicine GmbH, Gräfelfing, Germany) and the Actifit meniscus implant (Orteq, London, UK). The purpose of these resorbable scaffolds is to promote the in-growth of meniscal tissue, ultimately resulting in the regeneration of a meniscus formed by host tissue [1,86]. The collagen meniscus implant consists of a porous collagen matrix made primarily of purified type I collagen derived from a bovine Achilles tendon, with small amounts of glycosaminoglycan. Short- and long-term follow-up have both reported positive radiographic and clinical outcomes, including enhanced knee function, increased sports participation, and reduced pain. This treatment method also shows better results compared to partial medial meniscectomy [86]. The Actifit meniscus implant is a gradually degrading polymer scaffold made of polycaprolactone and urethane. Arthroscopy performed one year after surgery revealed successful integration of the implant in most patients. Moreover, six months after Actifit scaffold implantation, patients with irreparable medial and lateral meniscal defects showed notable improvement in pain and activity scores, compared to their baseline levels. However, designing implants could be challenging, especially in selecting a material that would match the properties of the native meniscus [1].

### 7.8. Mesenchymal Stem Cells

In addition to the previously discussed methods for treating meniscus injuries, the field of meniscus tissue engineering has been advancing rapidly in recent years. Tissue engineering leverages cells with regenerative potential to augment the healing of meniscal injuries. Research has focused on various cell types, including articular chondrocytes, meniscal fibrochondrocytes, and mesenchymal stem cells (MSCs) [87]. In orthopedics, bone marrow is the primary source of MSCs, but alternative sources include the synovial membrane, adipose tissue, meniscus-derived MSCs, and extra-articular tissues, like the dermis [87,88].

Autologous meniscal fibrocartilage cells harvested from meniscectomized debris are theoretically ideal seed cells for meniscal regeneration. These cells naturally possess the appropriate cellular phenotype and enable autologous therapy, minimizing the risks of morbidity and immune rejection. Adult mesenchymal stem cells derived from the mesoderm could be even more effective for meniscal regeneration due to their ability to self-renew, differentiate into multiple lineages, modulate immune responses, and provide a home for injury sites [89].

Vangsness et al. conducted a clinical trial in which a group of fifty-five patients across seven institutions underwent partial medial meniscectomy. Within seven to ten days following the procedure, each patient received a single superolateral knee injection. They were randomly assigned to one of three treatment groups: Group A received 50 million allogeneic mesenchymal stem cells; Group B received 150 million allogeneic mesenchymal stem cells; and the control group received a sodium hyaluronate solution. Patients were monitored over a two-year period to assess safety, meniscus regeneration, overall knee joint health, and clinical outcomes. Follow-up evaluations included periodic magnetic resonance imaging (MRI). At twelve months post-meniscectomy, quantitative MRI showed a significant increase in meniscal volume (predefined as at least a 15% increase) in 24% of patients in Group A and 6% of patients in Group B. None of the patients in the control group reached this 15% threshold. Additionally, patients with osteoarthritic changes who were treated with mesenchymal stem cells reported a significant reduction in pain compared to those in the control group, as measured by visual analog scale (VAS) scores. These findings support the further investigation of human MSCs for their potential regenerative and protective effects on knee tissue [90].

Centeno et al.’s study presents a case report involving a consenting volunteer, in which mesenchymal stem cells were extracted and cultured ex vivo from bone marrow aspirated from the iliac crest. These cells were then percutaneously injected into the participant’s knee, which had MRI-confirmed degenerative joint disease. Clinical and radiographic changes were assessed through pre- and post-treatment evaluations, including subjective visual analog pain scores, physical therapy assessments, and MRIs. At 24 weeks post-injection, the patient showed statistically significant cartilage and meniscus growth on MRI, along with improved range of motion and reduced modified VAS pain scores [91].

The exact roles and mechanisms by which MSCs contribute to enhanced meniscus healing remain unclear. It is believed that MSCs improve meniscus healing through their paracrine functions, secreting various growth factors that may promote angiogenesis, cell differentiation, migration, and other regenerative processes [92].

Furthermore, existing studies are constrained by the absence of standardized outcome measures, which complicates comparisons between different approaches. Additionally, only a limited number of studies include mechanical testing of the regenerated menisci [93,94]. Various methods of administering MSCs are also possible. The most frequently mentioned method is injection [93,95]. We can also distinguish scaffold-based delivery [96] and local application, direct implantation, and tissue-engineered construct application [97].

As previously stated, it remains uncertain whether meniscus regeneration is driven by the direct action of mesenchymal cells or rather by the secretion of specific stimulating factors [98]. Although growth factors and mononucleated cells appear to enable meniscus regeneration, no cell-based approach has yet been adopted in clinical practice [99]. The main obstacles to implementing such strategies include regulatory restrictions and the requirement for cell expansion before transplantation, which significantly increases treatment costs [97].

There is a significant amount of preclinical research on the use of MSCs in meniscal regeneration, with numerous studies conducted in animal models. However, the in vivo application of MSCs for treating human meniscal injuries has been explored in only a limited number of studies [87,95]. Despite these limitations, repair of the human meniscus by MSCs has great potential in clinical treatment. Further clinical studies are required to elucidate the promising benefits of stem cells, as current evidence is insufficient to definitively recommend their use.

### 7.9. The Economic Feasibility of the New Treatments

The use of PRP injections is viewed as a potential method to alleviate pain and enhance joint function in patients with knee osteoarthritis, with the goal of postponing or avoiding surgical procedures. Clinics providing PRP therapy generally require patients to cover the costs out-of-pocket and actively market their services [100].

In the United States, PRP has become a widely used treatment for osteoarthritis, generating USD 93.4 million in market revenue in 2015, with expectations for continued growth in the upcoming years. However, due to a lack of high-quality evidence supporting its effectiveness, most insurance providers do not cover this treatment, leading patients to bear significant out-of-pocket expenses [101].

The estimated cost of a single PRP injection ranged from USD 200 to USD 500 in 2012, increasing to approximately USD 450 to USD 2500 per injection by 2015. Data from 179 centers across the U.S. suggest an average PRP procedure cost of USD 714 (95% CI: USD 691–USD 737). Despite these costs, the PRP industry has grown considerably over the past decade, though the current commercial pricing of this therapy remains uncertain [101].

On the other hand, mesenchymal stem cells (MSC), also referred to as bone marrow stromal cells, human marrow cells, mesenchymal progenitor cells, or multipotent adult stem cells, possess the ability to differentiate into cartilage, bone, and fat tissue. They also have an intrinsic capacity for self-renewal and rapid proliferation. However, MSC therapies are not covered by most insurance providers, and their costs remain high. For instance, in the United States, the cost of a single stem cell therapy session for osteoarthritis treatment has been estimated at USD 5156 (95% CI: USD 4550–USD 5762) based on data from 273 centers [101].

## 8. Complications Following Meniscus Treatment

While the aforementioned methods are highly effective, each carries the risk of complications that may impact the recovery process and the long-term function of the knee joint.

### 8.1. Complications After Arthroscopy

Arthroscopy is generally regarded as a safe treatment method; however, it can lead to various complications, including joint infections, deep vein thrombosis (DVT) and pulmonary embolism (PE), compartment syndrome, hemarthrosis, and nerve and blood vessel injuries.

Joint infections are a rare complication of arthroscopy; however, when they occur, they can lead to septic arthritis, bacteremia, and osteomyelitis. Treatment options include arthroscopic debridement and irrigation, arthrotomy, and repeated knee aspirations. These procedures are combined with intravenous antibiotic therapy after the identification of the pathogen. The key objectives are decompression of the knee joint, removal of destructive proteolytic enzymes produced by bacteria, and complete eradication of the infection [67].

Deep vein thrombosis (DVT) and pulmonary embolism (PE) are associated with prolonged immobilization and reduced blood circulation. They are common and clinically significant complications of major surgical procedures. In the case of arthroscopy, this is because many institutions do not provide perioperative thromboprophylaxis for patients undergoing same-day surgery, believing that these procedures are less invasive and allow for a quicker return to normal physical activity. To minimize this risk, it is crucial to ensure appropriate thromboprophylaxis [67,102].

Compartment syndrome is caused by excessive pressure from fluids used during arthroscopy. Similar to chronic exertional compartment syndrome (CECS), treatment options typically include conservative approaches such as rest, physical therapy, and activity modification. If these measures fail to provide relief, surgical fasciotomy may be necessary. In cases of suspected acute compartment syndrome immediately following arthroscopy, urgent surgical intervention may be required to relieve pressure within the muscle compartments [67,103].

Nerve and blood vessel injuries can result in sensory impairment and chronic pain. The risk of complications stems from the close proximity of blood vessels, nerves, and tendons, which surgeons should carefully consider. There is no clear consensus on postoperative rehabilitation. Weight-bearing in extension is unlikely to be a critical factor in typical longitudinal injuries. However, greater degrees of flexion, especially under load, can cause significant meniscal displacement and shear forces, necessitating a cautious approach [67,98].

Hemarthrosis (bleeding into the joint) may cause swelling and limited range of motion. This risk stems from the use of anticoagulant medications, which are essential for preventing embolism and thrombosis. Therefore, conservative management is crucial, including joint immobilization, cooling, and limiting weight-bearing. Once the acute phase resolves, rehabilitation exercises are introduced to restore mobility and muscle strength. However, in cases of significant blood accumulation, joint aspiration may be necessary for its removal [67,104].

### 8.2. Complications After Meniscectomy

The most common complication of meniscectomy is an increased risk of degenerative changes. Partial or complete removal of the meniscus disrupts the even distribution of forces within the joint, accelerating cartilage degeneration. Research has consistently shown that meniscectomy raises the risk of osteoarthritis compared to cases where the meniscus remains intact or is repaired. This correlation is attributed to the loss of meniscal function and resulting alterations in knee biomechanics [4,69].

One of the major risk factors for OA is meniscectomy (Mx), which causes rapid and progressive OA [105]. Repairing medial meniscus root tears results in less osteoarthritis compared to total meniscectomy or nonsurgical treatment and is a more cost-effective option. Although small-scale randomized clinical trials directly comparing these approaches are needed for further confirmation, the current evidence strongly supports meniscus repair as the preferred initial treatment for medial meniscus root tears.

Over 10 years, meniscus repair, meniscectomy, and nonoperative treatment led to 53.0%, 99.3%, and 95.1% rates of osteoarthritis and 33.5%, 51.5%, and 45.5% rates of total knee replacement, respectively. Meta-analysis confirmed lower osteoarthritis and total knee replacement rates for meniscus repair versus meniscectomy and nonoperative treatment. From a cost-effectiveness perspective, the cost of meniscus repair was the lowest in 10 years at USD 22,590 compared to USD 31,528 for meniscal removal and USD 25,006 for nonoperative treatment. It also produced the highest projected quality adjusted life years (QALYs) of 6892, 6533, and 6693, making it an economically dominant strategy. Even with different assumptions, meniscus repair remained either cost-effective or predominant compared to alternative treatments as early as five years after surgery (Table 3) [106].

This suggests that, from both a clinical and economic standpoint, prioritizing meniscal repair, when possible, may provide the best long-term value. However, patient-specific factors and the feasibility of surgery should be taken into account when making decisions [106].

Due to the risks associated with meniscectomy, there is increasing emphasis on meniscus-preserving techniques to slow degeneration and maintain joint function. Research suggests that lateral meniscectomy, in particular, carries a greater risk of severe complications compared to medial meniscectomy. One of the most concerning outcomes is rapid chondrolysis, especially in young athletes. This condition is characterized by persistent joint pain and effusion, with early X-rays in the Schuss view or under load in semi-flexion often revealing significant joint space narrowing [10].

Treatment approaches such as discontinuing sports activities, administering corticosteroid injections, and performing arthroscopic joint lavage in cases of persistent effusion may help alleviate symptoms. However, these interventions frequently come at the cost of significant cartilage damage, underscoring the need to prioritize meniscus-preserving alternatives whenever possible [10].

### 8.3. Complications After Meniscus Suturing

Complications following meniscus suturing can include various issues related to both the healing process and potential injuries to surrounding structures. One of the most common complications is failed healing, particularly in tears located in the avascular zone of the meniscus (white-white), where the lack of blood supply significantly impairs tissue regeneration [72,107].

Patients may also experience pain at the site where sutures are tied over the joint capsule, especially with the inside-out technique. This discomfort typically resolves on its own as the absorbable sutures break down. Another significant complication is the risk of neurovascular injury, which is highest with the inside-out and outside-in techniques, particularly when suturing the posterior portion of the meniscus. In such cases, the all-inside technique may help reduce this risk [72,107].

Additionally, improper meniscus healing can lead to the formation of a parameniscal cyst. Though rare, postoperative joint infections can also occur, but proper aseptic measures help keep this risk low. Another potential issue is inadequate stabilization of the meniscus, which may result in re-injury and require further surgical intervention [72,107].

Achieving the best possible outcomes depends on selecting the appropriate suturing technique, conducting a thorough preoperative assessment, and ensuring careful postoperative management [72,107].

### 8.4. Complications Associated with the Use of Platelet-Rich Plasma (PRP)

Despite its many advantages, PRP therapy may have certain side effects. Patients may experience pain, along with swelling and irritation at the injection site, which are typically mild and subside on their own. In rare cases, an improper aseptic technique can lead to infection. Some patients may also have a temporary flare-up of pain and inflammation following the injection. Furthermore, the lack of a standardized PRP preparation method can lead to variations in treatment effectiveness, resulting in inconsistent outcomes [80].

### 8.5. Complications Associated with the Use of Synthetic Implants

The implantation of a collagen meniscus implant (CMI) carries the risk of complications and may require reoperation. The most common issues include joint swelling and pain, which can occur after the procedure. In some cases, difficulties with wound healing, inflammation, infections, or joint instability may also arise [85].

While most complications are mild and resolve naturally or with appropriate treatment, some cases may require additional surgical interventions. The most common reoperations include joint debridement, implant removal, and procedures to improve knee stability [85].

In summary, although CMI can offer significant benefits in terms of joint function and pain relief, the potential risk of complications should be carefully considered when making treatment decisions.

## 9. Optimal Treatment Choice for Meniscal Injuries

Selecting the appropriate treatment for meniscal injuries depends on multiple factors that influence healing potential and the long-term function of the knee joint. One of the most critical considerations is meniscal vascularization. Well-vascularized areas, known as the red zone, have a significantly higher regenerative capacity, making them more suitable for meniscal repair. In contrast, injuries in the poorly vascularized white zone have limited potential for spontaneous healing, often necessitating partial meniscectomy [72].

The type of meniscal tear also plays a crucial role in determining the treatment approach. Longitudinal vertical tears in the red zone are typically good candidates for repair, whereas radial, horizontal, and complex tears, especially those in avascular regions, have limited regenerative capacity and often require surgical intervention [72].

Patient age has traditionally been viewed as a limiting factor for meniscal repair due to the reduced regenerative capacity of tissues in older individuals. However, recent studies suggest that the procedure can still be effective in patients over 40, particularly when other favorable factors—such as knee stability and a suitable tear type—are present [72].

Knee stability is another key determinant of treatment success. Meniscal repair generally yields better outcomes when the knee is stable. In cases of instability, such as those involving an anterior cruciate ligament (ACL) injury, treatment effectiveness may improve if ACL reconstruction is performed concurrently. This combined approach enhances meniscal healing by promoting the release of growth factors and stem cells during bone tunnel drilling, which supports tissue regeneration [72].

The patient’s level of physical activity is also an essential factor. Physically active individuals, especially athletes, may benefit more from meniscus-preserving techniques, as these help maintain meniscal function and reduce the risk of premature degenerative changes in the joint [72].

Additionally, research has shown that arthroscopic partial meniscectomy does not provide clinically significant benefits over exercise therapy for patients with degenerative meniscal injuries in the first two years following the procedure [108]. Katz et al. [109] reported that the risk of total knee replacement—used to treat end-stage knee osteoarthritis—was five times higher in patients who underwent surgery compared to those who chose exercise-based therapy. Similar findings were reported in a randomized clinical trial (RCT) by Julia C. A. Noorduyn et al. [108], which demonstrated that exercise-based physical therapy was non-inferior to arthroscopic partial meniscectomy in terms of patient-reported knee function over a five-year follow-up period. Both groups showed minimal and comparable progression of knee osteoarthritis.

These findings further support the recommendation that exercise-based physical therapy should be the first-line treatment for degenerative meniscal injuries, with meniscectomy reserved for patients experiencing persistent symptoms or severe mechanical dysfunction.

In conclusion, the choice of the optimal treatment for meniscal injuries should be tailored to each patient’s individual characteristics. The decision should be based on factors such as the location and type of tear, the degree of meniscal vascularization, patient age, knee stability, and activity level. While no universal treatment algorithm exists, considering these factors allows for a more informed and effective approach to therapy planning [72].

## 10. Conclusions

The menisci are crucial structures within the knee joint because they play an important role in load distribution, shock absorption, stability, lubrication, and proprioception. The menisci are subject to many types of injuries, which can lead to rapid degeneration of the knee joint. Their unique and complex structure makes treatment and repair challenging for both the patient and the surgeon. The potential for treatment depends mainly on the location of the lesion. Due to the limited healing capacity of the meniscus, currently used methods do not produce satisfactory results. At present, research is underway on new methods of meniscus tissue regeneration. Mesenchymal stem cells have been particularly intensively studied and show high regeneration potential, but there is not enough research to use them widely. Maintaining the characteristic composition and organization of the meniscus is crucial for the health of the knee joint. In spite of the advancements in understanding meniscal function and its preservation, much remains to be studied. Therefore, meniscus research should be directed towards modern regeneration methods that have better healing potential than currently used techniques.

## 11. Limitations

The main limitations of this study were the limited number of current papers that describe in detail the new methods of meniscus tissue regeneration. Moreover, another probable limitation of this study was that there is a limited number of current, relevant studies of which access to the full texts was gained. Regardless, we used the most recent literature, from which we extracted the most crucial and correct data for our paper.

Additionally, one of the primary challenges is the variability in patient outcomes, which can be influenced by factors such as age, activity level, and the extent of the injury. While techniques like meniscus repair, meniscus transplantation, and biologic therapies show promise, their long-term efficacy is still under investigation, and some patients may experience re-tears or incomplete healing.

Furthermore, while minimally invasive surgical techniques have improved recovery times and reduced complications, there is still a risk of postoperative pain, stiffness, and degenerative changes over time. More research is needed to optimize treatment protocols and identify the best candidates for each procedure. Finally, the lack of large-scale, long-term clinical trials makes it difficult to draw definitive conclusions about the superiority of one treatment method over another.

Future research should focus on advancing regenerative therapies such as stem cell treatment, PRP, and biomaterials to enhance meniscus healing and regeneration. Improved repair techniques, including stronger sutures, better fixation devices, and methods to heal avascular meniscus zones, are essential for reducing failure rates. Personalized treatment approaches using AI and predictive models can help tailor therapies based on patient-specific factors. Large-scale, long-term studies are needed to assess the durability of treatments and the progression of osteoarthritis.

## Figures and Tables

**Table 1:** Meniscus function summary.

Biomechanical Aspect	Description
Load Transmission	Meniscus carries 40–60% of the knee load (lateral: 65–70%, medial: 40–50%), increasing to 90% in flexion. A larger contact area reduces stress.
Shock Absorption	High water content enables shock absorption. Meniscectomy reduces this capacity by 20%, increasing osteoarthritis risk.
Stability	The lateral meniscus is more mobile, adapting to femoral motion, while the medial meniscus is more stable and acts as a secondary stabilizer.
Lubrication and Nutrition	Synovial fluid and proteoglycans (e.g., lubricin) reduce friction and aid joint function.

**Table 3:** Table showing a comparison of treatments in terms of efficacy, indications, contraindications, and risk of complications.

Treatment Method	Efficacy	Indications	Contraindications	Risk of Complications
Meniscus repair (inside-out, outside-in, all-inside)	79.5–100% (depending on the technique)	Young, active patients; longitudinal vertical tears in the red zone	Extensive tears in the white zone, advanced degenerative changes	Postoperative stiffness, nerve and vascular injuries, meniscal cyst formation
Partial meniscectomy	Short-term functional improvement, but increased risk of degenerative changes	Patients with chronic symptoms, non-repairable meniscus injuries	Young, active patients; injuries suitable for repair	Increased risk of osteoarthritis, chondrosis, joint instability
Meniscal scaffold with collagen implant (CMI)	Significant functional improvement and pain reduction	Patients with partial meniscectomy	Total meniscectomy, joint instability	Swelling, pain, wound healing issues, implant instability, risk of reoperation
Platelet-rich plasma therapy (PRP)	Short-term symptom improvement, supports healing	Adjunct to surgical treatment, supports healing after meniscus repair	Limited data on chronic injuries	Pain and swelling at the injection site, risk of infection, variable efficacy due to lack of standardization
Mesenchymal stem cell therapy (MSC)	Preliminary studies show meniscus tissue regeneration and pain reduction	Patients after partial meniscectomy, injuries in poorly vascularized areas	Lack of established clinical protocols, high treatment costs	Uncertain long-term efficacy, lack of therapy standardization, risk of immune reactions
