# Correction: Kuczyński et al. The Meniscus: Basic Science and Therapeutic Approaches. *J. Clin. Med.* 2025, *14*, 2020

**DOI:** 10.3390/jcm15218504

**Published:** 2026-11-02

**Authors:** Nikodem Kuczyński, Julia Boś, Kinga Białoskórska, Zuzanna Aleksandrowicz, Bartosz Turoń, Maria Zabrzyńska, Klaudia Bonowicz, Maciej Gagat

**Affiliations:** 1 Department of Morphological and Physiological Sciences, Faculty of Medicine, Collegium Medicum, Mazovian Academy in Płock, 09-402 Płock, Poland; n.kuczynski@mazowiecka.edu.pl (N.K.); j.bos@mazowiecka.edu.pl (J.B.); kingabialoskorska@gmail.com (K.B.); ale.zuz@wp.pl (Z.A.); klaudia.bonowicz@cm.umk.pl (K.B.); 2 Department of Trauma and Orthopedics, Regional Specialist Hospital in Grudziądz, 86-300 Grudziądz, Poland; b.turon@medipunkt.pl; 3 Department of Family Medicine, Faculty of Medicine, Collegium Medicum in Bydgoszcz, Nicolaus Copernicus University in Toruń, 85-094 Bydgoszcz, Poland; maria.zabrzynska@cm.umk.pl; 4 Department of Histology and Embryology, Faculty of Medicine, Collegium Medicum in Bydgoszcz, Nicolaus Copernicus University in Toruń, 85-092 Bydgoszcz, Poland

Error in Figures 1 and 2

In the original publication [1], the graphical presentation of Figures 1 and 2 required correction. During a post-publication review of the artwork, the authors identified that selected labels, leader lines, and anatomical boundaries were not displayed with optimal clarity at the final publication scale. The figures were therefore redrawn to improve image resolution, contrast, label placement, consistency of typography, and the visual identification of the relevant anatomical structures and meniscal attachment sites. These changes are limited to the graphical presentation and do not alter the anatomical content, interpretation, or scientific conclusions of the article. The corrected Figure 1 and Figure 2 appear below.

The authors state that the scientific conclusions are unaffected. This correction was approved by the Academic Editor. The original publication has also been updated.

## Figures and Tables

**Figure 1:**
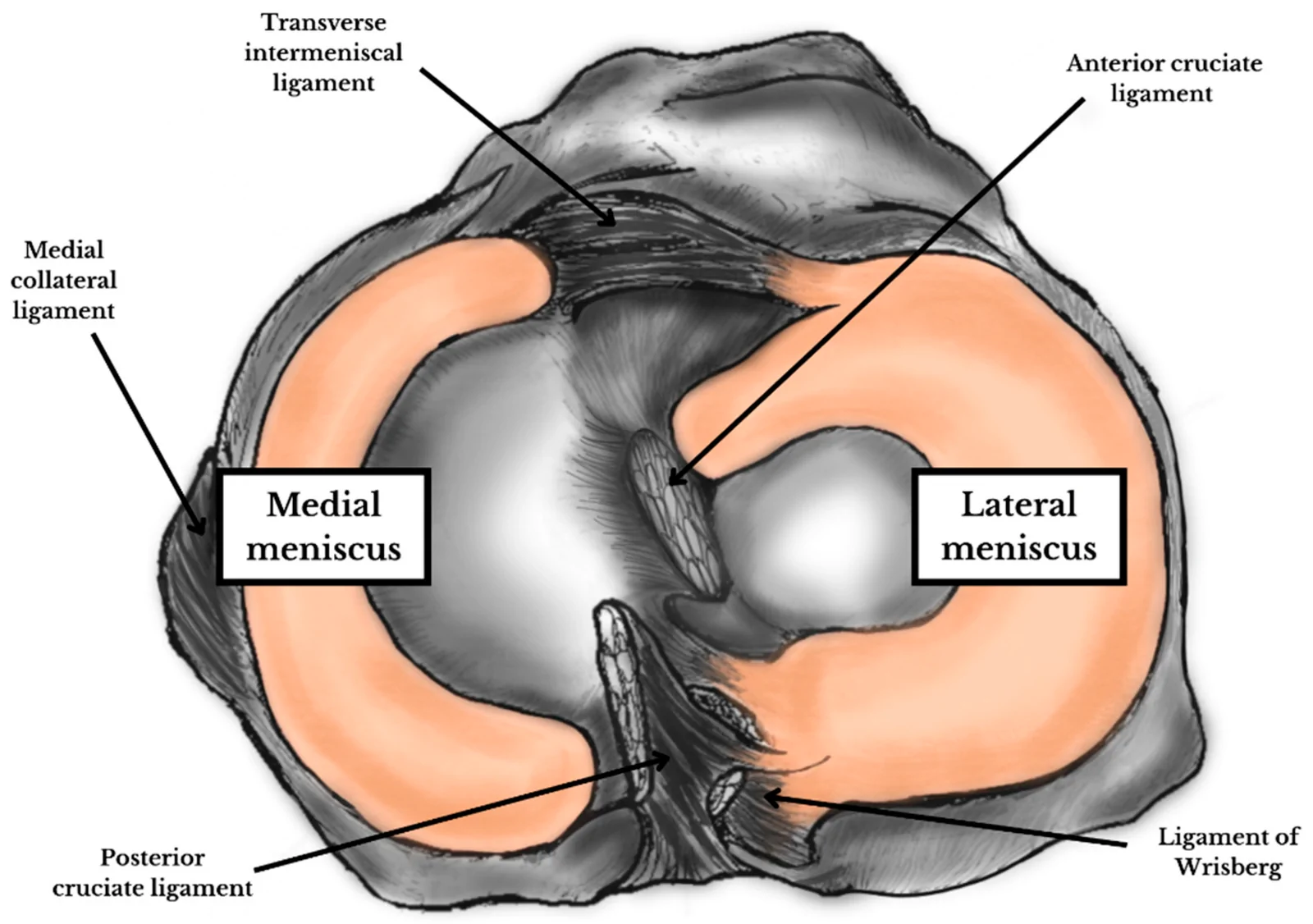
The anatomy of the meniscus viewed from above.

**Figure 2:**
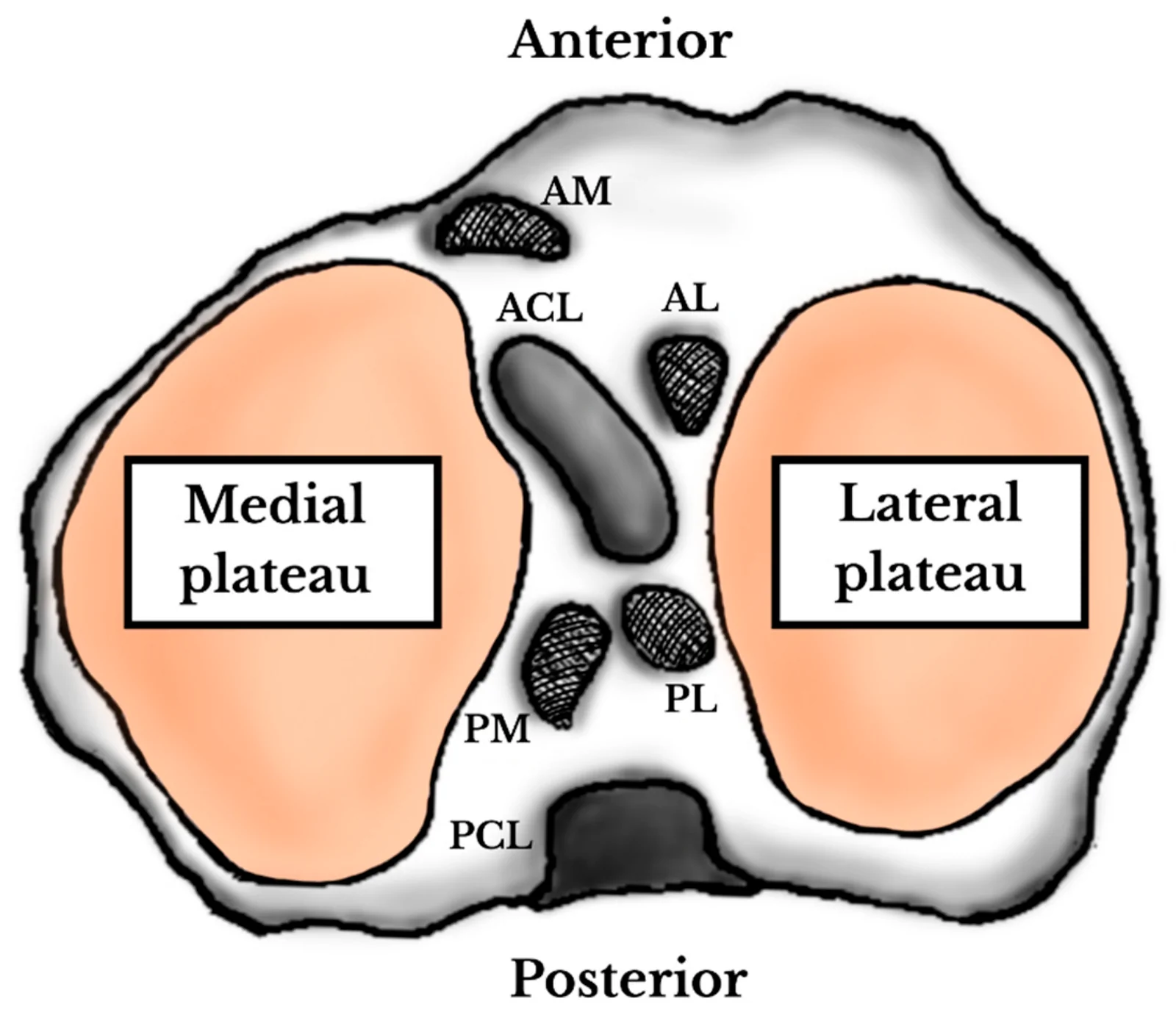
Meniscus horn insertion sites viewed from above. AL: anterior horn lateral meniscus; AM: anterior horn medial meniscus; ACL: anterior cruciate ligament; PCL: posterior cruciate ligament; PL: posterior horn lateral meniscus; PM: posterior horn medial meniscus.
